# To disconfirm or not to disconfirm: a null prediction vs. no prediction

**DOI:** 10.3389/fpsyg.2013.00733

**Published:** 2013-10-09

**Authors:** David Trafimow

**Affiliations:** Department of Psychology, New Mexico State UniversityLas Cruces, NM, USA

**Keywords:** null hypothesis, no prediction, failed prediction, accounting for data, does not account for data

As a researcher who reads journal articles, a journal reviewer, and a journal editor, I have lost track of the countless times I have encountered a particular type of scenario. An author obtains a statistically significant finding, points out that a particular theory fails to predict that finding, and argues that therefore the finding disconfirms the theory. But as I recently pointed out to a doubtless disappointed author in my rejection letter, this argument can be less strong than it appears at first blush. To see why, it is important to distinguish between two possibilities.

The first possibility is that the theory contains one or more propositions that, combined with reasonable auxiliary assumptions, really does lead to a particular prediction, whether of a result in a particular direction or a null effect. In that case, the failure of the prediction to materialize in the data really does constitute a problem for the theory. But there is a second possibility that often occurs. Specifically, a theory does not contain any propositions that lead to a prediction in the author's experimental paradigm. Rather the author's paradigm includes factors that are not mentioned in the theory. So the theory does not predict a null (or other) effect at all. Rather, the theory fails to make any prediction, whatsoever, with respect to the author's experimental paradigm. Naturally, when factors that are not included in the theory, but are included in the new paradigm, cause a statistically significant effect to occur, it is true that the theory “cannot account for the data.” But this is very different from saying that the data “are a problem” for the theory. The data are not a problem for the theory because the data are irrelevant to the theory. Unfortunately, authors, reviewers, and readers tend to interpret “cannot account for the data” as equivalent to “are a problem for the theory.” And so the data take on an unjustified level of importance.

It sometimes happens that a researcher feels that a theory ought to make a prediction in the experimental paradigm being used. But that the researcher feels this way does not make it so. Even if the researcher could make a convincing case that the theory ought to have included the researcher's variables, it would not be the finding itself that would militate against the theory but rather the argument that the theory failed to include everything that it should have included.

To avoid directly criticizing the guilty, let me make up an example involving the old frustration-aggression hypothesis that having a goal blocked causes frustration and the frustration causes aggression (see Berkowitz, 1989 for a review). Suppose a researcher uses an experimental paradigm where participants are bribed with money to be aggressive in the experimental condition but are not bribed in the control condition. The researcher observes more aggression in the experimental condition than in the control condition, and concludes the following: First, the frustration-aggression hypothesis fails to predict the effect. Second, because the effect happened, the frustration-aggression hypothesis is disconfirmed. The fallacy is that the frustration-aggression hypothesis does not predict a null effect, rather it fails to make any prediction whatsoever, and these are not the same thing. The reason the hypothesis does not make any prediction is because it concerns the blocking of goals whereas the experiment concerns the entirely different matter of financial bribery. Thus, the data do not disconfirm the frustration-aggression hypothesis; rather, the data are *irrelevant* to the hypothesis. In addition, that the researcher might feel like the frustration-aggression hypothesis should have included bribery does not mean that the finding is a problem for frustration-aggression hypothesis, though if the researcher were able to make a convincing argument about why the hypothesis should have included bribery, the argument (rather than the finding) might be problematic.

Why do psychology researchers so often commit this fallacy? My guess is that it stems partly from the dominance of the null hypothesis significance testing procedure (NHSTP). Although the NHSTP has been argued to be invalid (e.g., Trafimow, 2003), that is not my point here. My point here is that the NHSTP puts a premium on statistically significant effects. If a significant effect is obtained, the NHSTP allows the researcher to conclude that the null hypothesis of no difference is wrong, and this wrongness is interpreted as evidence in support of the author's hypothesis. In contrast, if the obtained effect is not statistically significant, the NHSTP does not allow the researcher to draw any conclusion other than the extremely weak one that he or she failed to reject the null hypothesis. This extremely weak conclusion does not convincingly disconfirm or support any substantive hypothesis. Consequently, psychology researchers are trained to try for statistically significant effects. But psychology researchers also are trained to try for theoretically meaningful effects. The easy way to combine these two considerations is to test one's theory against an alternative theory that does not make a prediction with respect to the researcher's paradigm, obtain a statistically significant effect, and interpret the data as disconfirming the alternative theory and supporting one's own theory. Of course, as I already have demonstrated, this sort of reasoning constitutes a fallacy because not making a prediction is not the same thing as predicting a null effect.

If psychology researchers somehow could have their minds wiped of their psychology training, the fallacy might be more obvious to them and less likely to be committed. But because of the necessity in our field to combine the rejection of the null hypothesis with the drawing of theoretically meaningful conclusions to publish manuscripts, the fallacy has become so ingrained as to become largely implicit and automatic. My goal is to make the fallacy explicit and to focus attention on it, so that it is not propagated into the next generation of psychology research.
